# Bioactive Hydrolysates from *Chlorella vulgaris*: Optimal Process and Bioactive Properties

**DOI:** 10.3390/molecules27082505

**Published:** 2022-04-13

**Authors:** Sara A. Cunha, Ezequiel R. Coscueta, Paulo Nova, Joana Laranjeira Silva, Maria Manuela Pintado

**Affiliations:** 1 CBQF—Centro de Biotecnologia e Química Fina—Laboratório Associado, Escola Superior de Biotecnologia, Universidade Católica Portuguesa, Rua Diogo Botelho 1327, 4169-005 Porto, Portugal; scunha@ucp.pt (S.A.C.); ecoscueta@ucp.pt (E.R.C.); pnova@ucp.pt (P.N.); 2 Allmicroalgae—Natural Products S.A., R&D Department, Rua 25 de Abril 19, 2445-287 Pataias, Portugal; joana.g.silva@allmicroalgae.com

**Keywords:** antioxidant, antihypertensive, enzymatic hydrolysis, alternative protein source, bioactive peptides, functional food, marine hydrolysates, sustainability

## Abstract

Microalgae have been described as a source of bioactive compounds, such as peptides. Microalgae are easy to produce, making them a sustainable resource for extracting active ingredients for industrial applications. Several microalgae species have interesting protein content, such as *Chlorella vulgaris* with around 52.2% of protein, making it promising for peptide hydrolysate production. Therefore, this work focused on the production of water-soluble hydrolysates rich in proteins/peptides from the microalgae *C. vulgaris* and studied bioactive properties. For that, a design of experiments (DOE) was performed to establish the optimal conditions to produce hydrolysates with higher levels of protein, as well as antioxidant and antihypertensive properties. Four experimental factors were considered (cellulase percentage, protease percentage, hydrolysis temperature, and hydrolysis duration) for three responses (protein content, antioxidant activity, and antihypertensive activity). The optimal conditions determined by the DOE allowed producing a scaled-up hydrolysate with 45% protein, with antioxidant activity, measured by oxygen radical absorbance capacity assay, of 1035 µmol TE/g protein, IC_50_ for angiotensin-converting enzyme inhibition activity of 286 µg protein/mL, and α-glucosidase inhibition of 31% (30 mg hydrolysate/mL). The obtained hydrolysates can be used as functional ingredients for food and nutraceuticals due to their antioxidant, antihypertensive, and antidiabetic potential. Moreover, the antioxidant potential of the extracts may be relevant for the cosmetic industry, especially in antiaging formulations.

## 1. Introduction

Microalgae are photosynthetic organisms with a rapid growth rate, which do not need cultivable land or freshwater for cultivation [1]. Furthermore, due to their CO_2_ sequestration capacity [2], industrial wastes and industrial produced CO_2_ can be targeted for microalgae cultivation. Microalgae have been explored as an alternative bioenergy source due to the decreased cost, renewability, and environmental benefits compared to the common sources [1]. However, their richness in interesting compounds, such as polysaccharides, lipids, proteins, and vitamins [3], highlights their potential for other industrial applications, such as biomedical, food, nutraceutical, and cosmetics. Microalgae use is very different across industries, such as, from higher to lower volume used, energy and bioremediation, chemical and materials, food and feed, and personal care and pharmaceutical industries. However, the value of the microalgae products developed appears in the inverse order, with the personal care and pharmaceutical industries being those producing the most valuable microalgae products [4]. The algae products market is currently estimated to be 4.7 billion USD and is expected to reach 6.4 billion USD by 2026, with a compound annual growth rate of 6.3% [5]. Asia, the United States, and Oceania are the main markets for algae manufacturing products, while Europe is responsible for just 5% [2].

*Chlorella vulgaris* is a microalga with an interesting nutritional composition, including lipids, carbohydrates, fiber, vitamins, and proteins [6]. *Chlorella* is especially interesting due to its protein content, which is usually up to 60% [7]. Hydrolyzing microalgae proteins can enhance several other interesting characteristics, as it leads to the release of bioactive peptides with several possible bioactivities. Several bioactive peptides have been produced from *C. vulgaris*, showing interesting properties such as antioxidant, antihypertensive, anti-inflammatory, anticancer, and antimicrobial [8]. Furthermore, microalgae peptides may also be of great interest due to their functional properties such as solubility, as well as emulsifying and foaming properties, which could be beneficial for industrial application [9].

Despite the high protein content, one of the main problems is accessing the intracellular proteins, since the microalgal cell wall is rich in cellulose, β1-3 glucan, proteins, lipids, ash, and glucosamine [10]. These compounds make it rigid and difficult to digest and, consequently, limit the extraction of proteins and peptide release. Therefore, it is important to weaken or break the cell wall to achieve a more efficient peptide extraction. Mild methods are always preferred so that proteins are not compromised. However, they are not always very efficient in protein release [11]. For that, mechanical methods appear to be the most effective in breaking the cell wall and releasing proteins, with bead milling and high-pressure homogenization being the most described methods for achieving a high protein release. However, it is necessary to have specific equipment to apply these high-energy-consuming techniques. On the other hand, chemical and enzymatic hydrolysis are considered simpler, more sustainable, and cheaper [11]. Furthermore, due to the rigidity of the cell wall, not all proteins will be bioavailable when consuming the intact microalgae. Thus, microalgal protein hydrolysates will increase protein/peptide/amino-acid bioavailability and, consequently, increase their value as an ingredients for food or nutraceutical applications [12]. Accordingly, consuming *C. vulgaris* hydrolysates may promote a higher protein/peptide/amino-acid absorption combined with several possible benefits for human health [8].

Antihypertensive and antioxidant hydrolysates may have a high interest for industrial applications. Hypertension is considered one of the major causes of cardiovascular diseases (CVDs). Several treatment drugs act by inhibiting angiotensin-converting enzyme-I (ACE-I) levels, resulting in a decrease in ACE-II, a strong vasopressor, and consequently upregulating the metabolism of bradykinin, a vasodilator [13,14]. These drugs are generally associated with side-effects [15]; thus, the development of new compounds able to inhibit ACE-I enzyme may have potential for pharmaceutical industries, as well as for food and nutraceuticals, since they can be used as a preventive product. Antioxidant hydrolysates stand out as some of the most interesting, with a higher potential for commercialization. We are frequently exposed to external factors that lead to the production of free radicals on our body [16], not to mention that, internally, we are constantly producing free radicals; moreover, due to several factors, such as aging, our body capacity to fight these free radicals is decreased [17]. Free radicals may lead to damage and/or alterations in cells, DNA, lipids, and proteins, among other vital molecules, resulting in diseases [18]. Thus, the development of functional food with antioxidant properties may help fight free radicals and improve the life quality of consumers. Furthermore, incorporation of antioxidant hydrolysates into cosmetic formulations may help to protect the skin from the generated free radicals, thus delaying skin aging.

This work focused on producing water-soluble hydrolysates rich in proteins and bioactive peptides from the microalga *C. vulgaris* by developing an optimal method based on an acid pretreatment followed by two sequential enzymatic hydrolyses, aiming to obtain high protein content and high bioactivities, namely, antioxidant and antihypertensive properties. Therefore, this work intended to obtain optimized microalgal hydrolysates, thus valorizing these species as a source of proteins and bioactive peptides.

## 2. Results

### 2.1. Optimization of the Production of Chlorella vulgaris Hydrolysates

#### 2.1.1. Acid Pretreatment and Enzymatic Hydrolysis

The microalga *C. vulgaris* was characterized regarding its nutrient and pigment composition, showing an interesting nutrient profile with protein, fat, carbohydrate, and dietary fiber contents of 52.2, 7.9, 10.9, and 15.5 g/100 g, respectively (Table 1). *C. vulgaris* is also an appealing chlorophyll source, with about 1533 mg/100 g. Due to its protein percentage, *C. vulgaris* is described as a source of bioactive peptides. However, microalgae have a rigid cell wall, making it difficult for proteases to access the intracellular proteins. Therefore, to overcome this problem, it is necessary to develop strategies to weaken the cellular wall and enhance protein availability. For that, before protease use, we evaluated the effect of a pretreatment with acid and a hydrolysis with a cellulase on protein release.

These preliminary studies showed that a pretreatment of the microalgae with 2% acetic acid solution could facilitate protein release when submitted to the subsequent enzymatic hydrolysis steps. We studied cellulase due to its ability to act on the microalgal cellular wall, degrading cellulose and making it easier to release proteins. Consequently, proteins become more available for the protease action, resulting in more peptide release. Our initial studies showed that combining the acid pretreatment with cellulase hydrolysis led to higher protein release.

The ratio of acid and cellulase, hydrolysis time, and temperature were previously tested. For the acid, the best conditions were a pretreatment with 2% acetic acid (*w*/*v*) for 1 h at 50 °C. For the hydrolysis with cellulase, incubating at 50 °C (according to the enzyme manufacturer’s indications) for 2 h was shown to be enough for protein release.

#### 2.1.2. Optimal Conditions

The sequential use of acid pretreatment and cellulase hydrolysis showed an effect on protein release. However, to achieve bioactive properties, a protease must be used to breakdown proteins into peptides. Furthermore, several factors can interfere with protein release and breakdown, as well as hydrolysate bioactive properties. Thus, the use of a design of experiments (DOE) may allow producing hydrolysates rich in proteins and peptides with optimized bioactivities. A Box–Behnken experimental design was created and analyzed using Statgraphic centurion software. The design resulted in 27 factor combinations tested in duplicate, resulting in 54 tests. The DOE matrix and the results obtained for the dependent variables are presented in Table 2.

Regarding hydrolysate production yield (Supplementary Materials), the lower mean values obtained were 47.00% ± 0.13%, 47.31% ± 0.09%, and 47.52% ± 0.12%, all observed for runs with a protease hydrolysis temperature of 50 °C and 1.67% protease (runs 6, 13, and 14). As for the three higher yields, they were verified for runs at 50 °C and central or high protease levels (runs 7, 24, and 26), with mean values of 76.02% ± 0.02%, 73.35% ± 0.06%, and 74.54% ± 1.20%. After determining the hydrolysates’ protein content, we calculated their correlation with the protein release, considering the microalgal protein content of 52.2% and the total biomass used. The lower protein release values were 55.65% ± 0.32%, 50.63% ± 0.10%, and 45.89% ± 0.25% (runs 2, 13, and 14), all tests held with the lower protease percentage tested, while the higher mean values were 68.12% ± 0.12%, 79.42% ± 0.24%, and 67.76% ± 0.42% (runs 4, 8, and 21), obtained for runs with protease hydrolysis held at 40 or 60 °C, and with central or higher levels of cellulase percentage.

**Figure 1:**
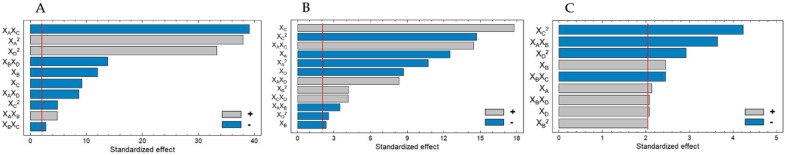
Recalculated Pareto charts with the effect of four experimental factors, in decreasing order, obtained for protein percentage (**A**), as well as antioxidant (**B**) and antihypertensive (**C**) properties, in the experimental design, showing the most influent factors. The vertical lines in the Pareto charts represent the significance level (*p* = 0.05). X_A_—temperature; X_B_—cellulase; X_C_—protease; X_D_—hydrolysis time.

**Figure 2:**
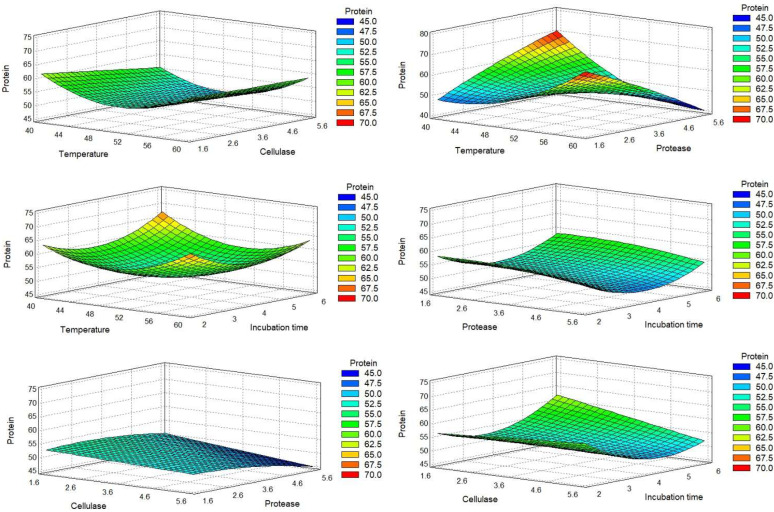
Response surfaces obtained for the protein release. Each response corresponds to the combined effect of the variations of two experimental factors, keeping the other two factors at their central level, on the protein release.

**Figure 3:**
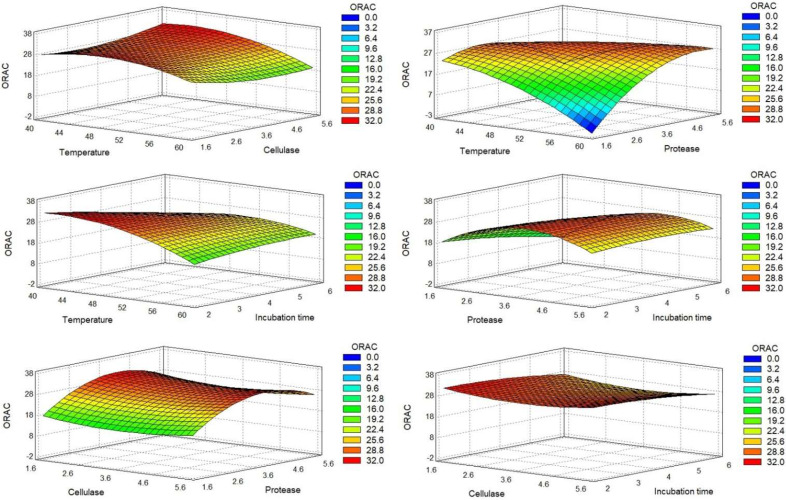
Response surfaces obtained for the antioxidant activity (ORAC). Each response corresponds to the combined effect of the variations of two experimental factors, keeping the other two factors at their central level, on the antioxidant activity.

**Figure 4:**
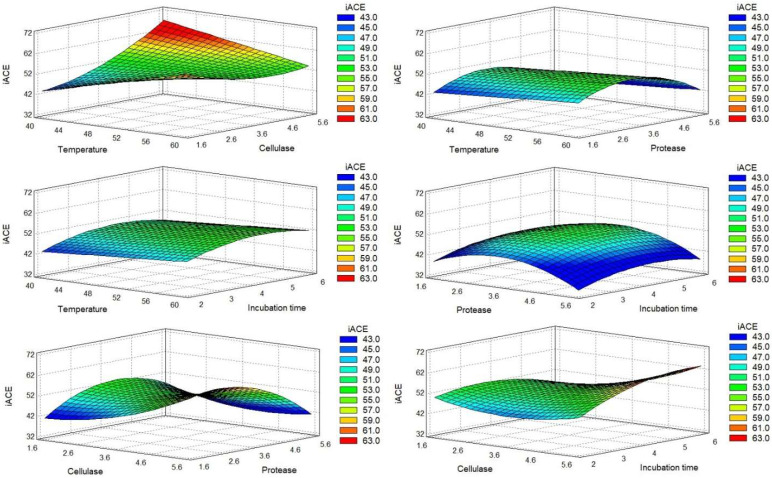
Response surface obtained for the inhibition of ACE (iACE). Each response corresponds to the combined effect of the variations of two experimental factors, keeping the other two factors at their central level, on the iACE.

For a more straightforward analysis, we express the DOE results graphically. The Pareto charts (Figure 1) allowed us to quickly identify the estimated significant coefficients of each factor with a higher effect in the model of each response. The surface response graphics are presented in Figure 2, Figure 3 and Figure 4.

The protein percentage (dry basis) of each run was evaluated by the Kjeldahl method. The results indicated that the sequential hydrolysis steps allowed the production of hydrolysates with protein content in the range of 47.62% to 84.46%. The recalculated Pareto charts showed that the quadratic effects of temperature and time (X_A_^2^ and X_D_^2^) appeared as the main influences, along with the interaction between temperature and protease. The linear effect of temperature and hydrolysis time did not influence protein release, thus meaning that protein percentage was similar in terms of the maximum and minimum values of these factors. The effect of X_A_^2^ and X_D_^2^ corroborates the previous statement, showing that the protein percentage was lower when temperature and hydrolysis time were closer to the central value; thus, more favorable conditions were found for extreme values. The interaction between temperature and protease (X_A_X_C_) was the most important factor mediating protein percentage, indicating that protein release was higher when temperature and protease were close to their minimum values. Regarding both enzymes used, their linear effect (X_B_ and X_C_) showed an inverse effect on protein release, indicating that protein percentage decreased when the enzyme percentage increased. Thus, higher protein content may be achieved with lower values of enzymes. For protease, its quadratic effect (X_C_^2^) showed that a higher protein percentage was achieved with intermediate protease values.

The final adjusted model is presented in Equation (1). Table 3 shows that the adjusted model explained 98.04% of protein percentage variability (*R*^2^).

Protein % = 132.971 − 5.0764 X_A_ − 0.202681X_B_ + 35.1809X_C_ − 4.96679X_D_ + 0.074295X_A_^2^ + 0.0659997X_A_X_B_ − 0.660452 X_A_X_C_ − 0.0929X_A_X_D_ − 0.201601X_B_X_C_ − 0.824626X_B_X_D_ − 0.339506X_C_^2^ + 1.54507X_D_^2^. (1)

The antioxidant activity of the hydrolysates was measured by the oxygen radical absorbance capacity (ORAC) assay. ORAC values appeared in a range from 9.24 to 31.95 µmol TE/mL (Table 2). The analysis of the recalculated Pareto charts (Figure 1B) showed that all the linear coefficients affected ORAC values, with X_B_, X_D_, and X_A_ having an inverse effect. X_D_ and X_A_ had a considerably high impact, indicating that an increase in temperature and/or time would lead to the production of hydrolysates with lower antioxidant potential. Combining this information with the influence of their quadratic factors (X_A_^2^ and X_D_^2^), it is shown that ORAC values were increased for intermediate values of these factors. Regarding the adjusted Pareto chart, the most important factors for the antioxidant property were the linear and quadratic effects of protease (X_C_ and X_C_^2^). Together, they showed that increased protease resulted in higher ORAC values until its central percentage, but had the inverse effect for extremely high protease percentages. The interaction between temperature and protease (X_A_X_C_) also had a relevant influence, which, allied with X_A_ and X_C_ effect, indicated that higher protease percentage is more beneficial for ORAC at lower temperatures. The final adjusted model, without nonsignificant data, is presented in Equation (2), with *R*^2^ indicating that the model explained 85.08% of ORAC variability (Table 4).

ORAC = 21.8233 + 2.03867X_A_ + 0.347851X_B_ − 9.86063X_C_ − 10.9486X_D_ − 0.04632X_A_^2^ − 0.09945X_A_X_B_ + 0.525791X_A_X_C_ + 0.19837X_A_X_D_ + 0.634165X_B_^2^ − 2.32134X_C_^2^ + 0.595499X_C_X_D_ − 0.269216X_D_^2^. (2)

The antihypertensive potential of the hydrolysates was measured by the percentage inhibition of the ACE enzyme (iACE), in a concentration of 0.5 mg/mL. The recalculated Pareto chart showed (Figure 1C) that the linear effect of temperature, cellulase, and time directly influenced iACE, especially cellulase, meaning that hydrolysis performed with higher values of these factors was expected to produce hydrolysates with higher iACE. The linear coefficient of the protease (X_C_) did not show an influence, meaning that extreme values led to similar iACE results. However, X_C_^2^ was the main influencing factor, showing that the main differences were found near protease central values, with an increased iACE for the central value tested. The combination of the linear (X_D_^2^) and quadratic (X_D_^2^) effects of time showed that a longer incubation time was necessary for producing iACE peptides; however, the more promising iACE was reached for central values. However, the model was not very capable of explaining iACE variability (Table 5). We observed that iACE values varied from 32.64% to 63.59%. The difference between both runs’ conditions was only the protease percentage used, with higher values for a high protease level.

Through multiple regression, the predicted response for the iACE could be obtained using the model in Equation (3).

iACE = −64.6335 + 1.49833X_A_ + 14.2919X_B_ + 20.0332X_C_ + 5.94323X_D_ − 0.38445X_A_X_B_ + 1.07788X_B_^2^ − 1.54665X_B_X_C_ + 1.09725X_B_X_D_ − 2.23165X_C_^2^ − 1.06835X_D_^2^. (3)

A Derringer desirability analysis was performed to maximize the multiple responses of the studied factors [19]. Thus, a desirability of 0.77 was obtained, and the predicted optimal conditions were a hydrolysis with 5% cellulase and 3.9% protease, performed at 40 °C for 2.3 h (Table 6). For those conditions, it was expected to obtain hydrolysates with 64% protein, an ORAC value of 32.13 µmol TE/mL, and an iACE of 51.81%.

### 2.2. Scaled-Up Hydrolysates Bioactivities

**Figure 5:**
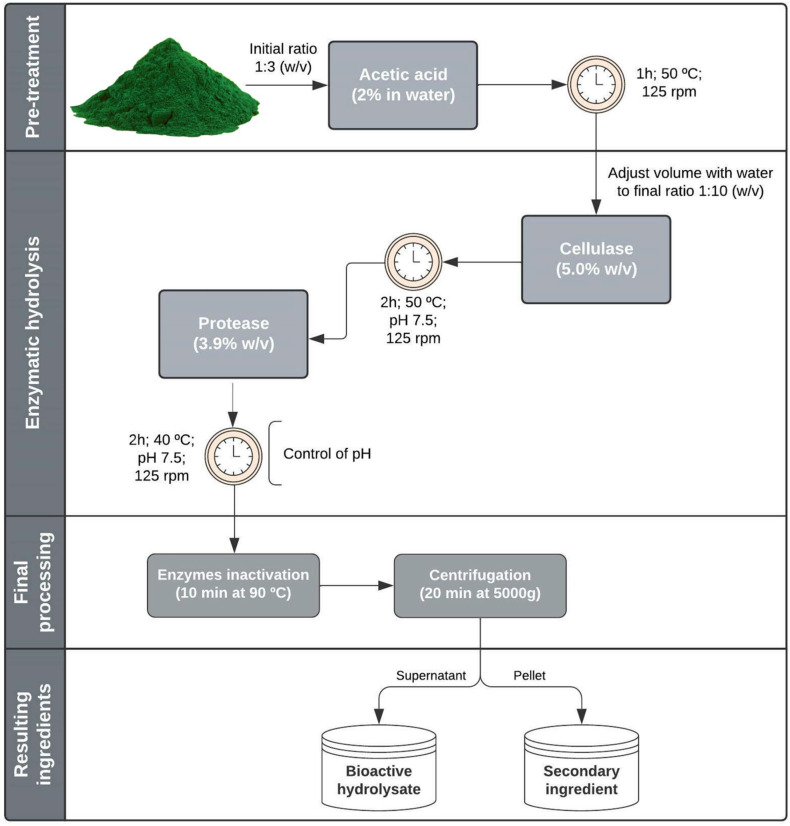
Representative scheme of the methodology performed for the production of bioactive hydrolysates.

A scaled-up extraction was performed using the optimal conditions determined on the DOE, maintaining the microalga–solvent ratio, but increasing it by 30 times. The scale-up extraction was performed in triplicate, using the preliminarily established parameters and the optimized enzyme percentages, temperature, and time. Thus, to produce bioactive hydrolysates from *C. vulgaris*, the methodology developed started with an acid hydrolysis using 2% acetic acid for 1 h at 50 °C. Secondly, the microalga was hydrolyzed with 5% cellulase for 2 h, at 50 °C. The last hydrolysis step was conducted with 3.9% of protease for 2 h, at 40 °C. The optimized methodology is schematized in Figure 5.

The scaled-up hydrolysis procedure resulted in a yield of 61% ± 0.5% on the production of the bioactive hydrolysate (the soluble fraction). This optimized hydrolysate showed 45% ± 1.7% protein content, corresponding to a release of 52.37% ± 0.36% of the total protein of the biomass used.

Regarding the bioactivities analyzed, the hydrolysate showed 463 ± 39.9 µmol TE/g hydrolysate or 1035 ± 68.7 µmol TE/g protein on ORAC, an IC_50_ for iACE of 286 ± 55.0 µg of protein/mL, and the capacity to inhibit 31% ± 3.9% of α-glucosidase activity (30 mg hydrolysate/mL) (Table 7).

### 2.3. Hydrolysate Protein/Peptide Profile

**Figure 6:**
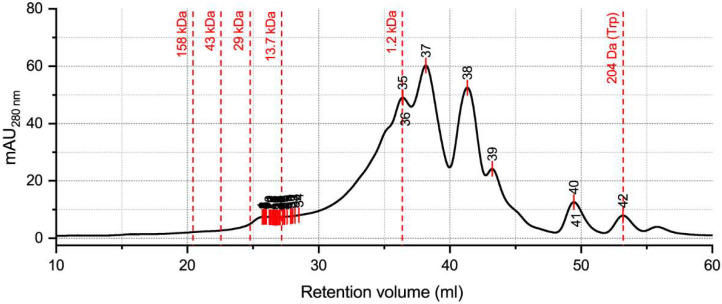
Protein and peptide profile by the size of molecular weight of *C. vulgaris* hydrolysate produced in the scale-up with the optimized conditions, showing the main MW ranges, the area of the main peak, and the localization of all the 42 identified peaks.

We analyzed the protein/peptide profile of the optimized hydrolysate by size exclusion chromatography (SEC). To identify hidden peaks due to overlapping, we applied the model local maximum, with a positive peak direction and a peak minimum height of 10%. This analysis allowed the identification of 42 peaks (Figure 6) (Supplementary Materials).

The local maximum analysis showed the existence of 42 peaks, with 35 peaks being located between retention volumes of 25 and 30 mL. The higher MW present in the hydrolysate corresponded to 19.54 kDa. The remaining 34 close peaks had MW between 18.86 and 6.88 kDa. However, the most abundant peptides had MW equal to (peaks 35 and 36) or lower than 1.2 kDa (peaks 37 to 42). The smallest MW peak was found to be 204 Da (peak 42).

## 3. Discussion

*C. vulgaris* has a very appealing protein content not only for direct use in the food industry, as a source of proteins, but also to produce bioactive peptides from these proteins. However, *C. vulgaris* has a rigid cell wall, and only about 20% of proteins are found bound to the cellular wall [20]. Therefore, to effectively produce peptides from *C. vulgaris* proteins, the rigid cell wall must be weakened. Our preliminary studies showed that an acid pretreatment, with acetic acid, before the enzymatic hydrolysis allowed better protein release from the microalgae *C. vulgaris*. The choice of acetic acid was based on two premises: first, it is a weak acid easily available for academic and industrial laboratories; second, it is generally recognized as safe for food use by the Food and Drug Administration [21].

In the proposed methodology, we used a low acid concentration, since it showed a positive effect on protein release, without compromising further steps. The sequential use of the acid pretreatment followed by cellulase hydrolysis was the most relevant strategy for protein release. The acid weakens the cell wall and facilitates the degradation of cellulose by cellulase, leaving the internal protein exposed. The subsequent hydrolysis, with the protease, is the most important stage for peptide production since the protease will break down the previously released proteins, transforming them into peptides.

Until now, no studies have used a sequential acid pretreatment followed by cellulase and subtilisin (a protease) hydrolysis to produce bioactive hydrolysates from *C. vulgaris*. The most used enzymes for peptide production from microalgae appear to be pepsin, trypsin, alcalase, papain, and flavourzyme [8]. The microalgal acid pretreatment was studied for bioethanol production, and another study showed that both sulfuric acid and acetic acid may be used as pretreatment for the release of fermentable sugars [22], but not protein release. For that, alkaline pretreatment is more often used in combination with mechanical methods [23,24].

The DOE allowed the optimization of a methodology for obtaining bioactive hydrolysates with high protein levels, as well as antioxidant and iACE potential. A high effect of protease percentage on the ORAC values was observed, with the higher response values occurring with central percentages of enzyme. Thus, the DOE showed that, until a certain level, a more hydrolyzed extract would produce smaller peptides with greater bioactivity. The iACE responses obtained on the DOE were not highly explained by the model. This may be explained by system variations or by a fast plateau being achieved [25].

The bioactive hydrolysates obtained, resulting from both the DOE and the scale-up, showed a green color, revealing the presence of pigments. Pigments, such as chlorophyll and carotenoids, are present within thylakoids, compartments found inside chloroplasts. Thus, the presence of pigments in the hydrolysates revealed that the chloroplast phospholipidic bilayer was breached. However, due to the hydrophobic nature of these pigments, it is possible that micelles were formed and/or there was cellular debris in the suspension unable to precipitate in the pellet, as suggested by other authors [20,26].

This methodology showed a yield of 61% for the bioactive hydrolysate production. It was expected that not all the microalgal biomass would be transformed into soluble hydrolysate due to the existence of cellular debris and hydrophobic compounds. However, it is important to highlight that the secondary ingredient produced (the insoluble fraction) was composed of lipids, polysaccharides, pigments, and the remaining proteins. Moreover, the cells remaining in this fraction were weakened and, thus, were expected to have a higher bioavailability of the intracellular compounds. Therefore, this secondary fraction may also be evaluated regarding its commercial value since it is likely that its nutritional composition could show benefits for human or animal health. Accordingly, it can be used as a nutritional complement for animal feed, as well as for enhancing human food products.

The scaled-up hydrolysate showed a water-soluble protein content of 45%, corresponding to a release of 52% of microalgal protein. These results are lower than those obtained in some DOE runs, which may be explained by the increased scale and different configurations of the reaction system. With some adjustments, these differences can be rectified, for example, by improving the stirring and the drying process. In scale-up, the stirring may have been insufficient to ensure full contact similar to DOE. On the other hand, the DOE runs were analyzed without prior lyophilization, whereas, in scaling, this process may have led to some protein aggregation (becoming insoluble). However, it is important to highlight that only around 20% of *C. vulgaris* proteins are found attached to the cellular wall [20]. Thus, the protein release percentage suggests that some intracellular proteins came out of the weakened membrane. Regarding protein release, the most effective methodologies described are mechanical ones, such as bead milling (96% of protein release) and high-pressure homogenization (66% of protein release) [20]. Nevertheless, these are techniques that require specific equipment. Despite having a lower protein release, the proposed method has an interesting value without resorting to mechanical or physical pretreatments.

The hydrolysate had a high bioactive potential, with mainly antioxidant and antihypertensive properties. ORAC, a reference method used in the food industry [27], was used to determine the antioxidant potential of the hydrolysates. The high obtained ORAC values indicate the great potential of this hydrolysate as an antioxidant ingredient. This value is higher than those found in the literature for *C. vulgaris* extracts produced using ultrasound-assisted extraction with water/ethanol as the solvent, with ORAC values of 33.07 and 29.35 µmol TE/g DW, with our values being closer to those obtained for the macroalga *Bifurcaria bifurcata* (537.38 and 575.02 µmol TE/g DW) [28]. Regarding iACE, our hydrolysate had high inhibitory ability, since we obtained an IC_50_ of 286 µg protein/mL, a lower IC_50_ than the reference value of 500 µg protein/mL [29]. The antidiabetic potential was also evaluated due to worldwide concerns about diabetes, associated with risk of premature death, vision loss, and heart attack, among others [30]. Few antidiabetic peptides have been described in algae, and no examples have been found in *C. vulgaris*. The cyanobacteria *Arthrospira platensis* and the macroalga *Porphyridium* sp. have already been studied for their effect on the insulin pathway or their ability to inhibit dipeptidyl peptidase IV (DPP-IV) enzyme [31]. In our work, we evaluated the ability of the hydrolysate to inhibit α-glucosidase, one of the main targets of type 2 diabetes treatment. Acarbose is one of the drugs used with action on this enzyme [32]; as such, we used it as the positive control. Flavonoids from the alga *Gracilaria corticate* showed an IC_50_ of 75 µg for α-glucosidase inhibition [33]. A *Chlorella pyrenoidosa* extract showed the ability to inhibit 68.28% of the enzyme activity at a concentration of 1000 µg/mL [34]. Thus, compared to other algal nonpeptide extracts, the obtained ability of 31% inhibition of α-glucosidase with 30 mg/mL of hydrolysate does not appear very promising as an α-glucosidase inhibitor. Table 8 shows a few examples of algae hydrolysates and extracts with described antioxidant, antihypertensive, or antidiabetic potential.

The protein and peptide profile showed the existence of 42 peaks. The hydrolysate showed a higher MW of 19.54 kDa and a smaller MW of 204 Da (free tryptophan). The area between 20 and 5 kDa showed the most peaks. However, regarding the chromatogram area, the more abundant peaks were those with MW around and lower than 1.2 kDa. *C. vulgaris* proteins have been described to have a MW range of 12–120 KD [20,39]. However, our hydrolysate did not show proteins with high MW. The higher content of peptides with MW < 1.2 kDa resulted from the hydrolysis steps to which the microalgae was submitted, especially the protease hydrolysis that led to an interesting protein breakdown and, consequently, the production of smaller peptides. The reported methodology led to efficient hydrolysis of the microalgal proteins, allowing the release of peptides with bioactive properties.

The produced bioactive hydrolysates, containing an interesting protein content, can be used as a natural amino-acid supplement. These hydrolysates may be especially promising for vegetarian and vegan consumers since they can be consumed directly or incorporated in food preparations to increase amino-acid intake from nonanimal origin. However, why would intact microalgae not be directly consumed as a source of protein? Hydrolysates have a much higher protein bioavailability than the intact microalgae, due to the complex and rigid nature of the cell wall. Thus, to achieve the same amino-acid absorption levels, lower hydrolysate intake may be needed. Furthermore, the hydrolysate presents bioactive properties that may contribute to health benefits for the consumer. However, for the development of functional foods, the previous principles should be confirmed by in vitro and in vivo analyses, to understand if bioactivities are maintained throughout the gastrointestinal tract [8]. In addition to the food industry, antioxidant ingredients are highly used in the cosmetic industry, mainly in the development of antiaging formulations. The mostly used antioxidants used are of synthetic origin. Thus, *C. vulgaris* antioxidant hydrolysates can be used as natural and sustainable alternatives instead of synthetic ones. For that, the hydrolysate compatibility with skin cell lines, such as fibroblasts and keratinocytes, should be evaluated, as well as the ability of the peptides to penetrate the skin barrier.

The present study presented a methodology allowing the production of two usable ingredients. The bioactive hydrolysate may be used in the personal care, nutraceutical, and food industries. The water-soluble nature of this hydrolysate, combined with its final powder format, makes it easy to incorporate into different matrices. On the other hand, the secondary ingredient may be incorporated into animal feed or even human food products. Furthermore, its potential as a cosmetic ingredient may be explored for formulations that are not entirely absorbed by the skin, such as peel-off masks. Therefore, we presented a sustainable and food-grade methodology to produce two final ingredients, aligned with a circular economy and working toward zero waste.

## 4. Materials and Methods

### 4.1. Materials

The enzymes used for microalgae hydrolysis, cellulase (New Cell Supreme 4000L) and subtilisin (a protease) (New Pro 16L), were supplied by Aquitex. The microalga was provided by Allmicroalgae-Natural Products, S.A (Pataias, Alcobaça). Sulfuric acid and Kjeldahl tablets (catalyst with 0.3% CuSO4 5H_2_O) were purchased from VWR Scientific (VWR chemicals, Karlsruhe, Germany); boric acid and hydrochloric acid (32%) were purchased from Merck (Darmdstadt, Germany); sodium hydroxide was purchased from LabChem (Zelienople, PA, USA). The proteins standard used (thyroglobulin, ferritin, aldolase, conalbumin, ovalbumin, carbonic anhydrase, ribonuclease A, and aprotinin) were acquired from GE Healthcare (Milwaukee, WI, USA), and the peptide KGYGGVSLPEW was obtained from GenScript (Nanjing, China).

Regarding the bioactivities, fluorescein, 2,2′-azo-bis-(2-methylpropionamidine)-dihydrochloride (≥97%), 6-hydroxy-2,5,7,8-tetramethylbroman-2-carboxylic acid (Trolox) (≥97%), angiotensin I-converting enzyme (EC 3.4.15.1, 5.1 U mg^−1^), and α-glucosidase (EC 3.2.1.20) were acquired from Sigma-Aldrich (St. Louis, MO, USA), while Abz–Gly–Phe (NO_2_)–Pro was supplied by Bachem Feinchemikalien (Bubendorf, Switzerland).

### 4.2. Hydrolysis Procedures

The microalga *C. vulgaris* was received in powder format (spray-dried). Preliminary studies were performed with different conditions to understand the limits to be established for the experimental design. For the experimental design, all reactions were performed with similar steps, only varying the tested factors. For each reaction, *C. vulgaris* was mixed with a solution of acetic acid (2% in deionized water) in a ratio of 1:3 (*w*/*v*) and incubated at 50 °C for 1 h. Secondly, deionized water was added until reaching a ratio of 1:10 (microalgae–water), and the pH was adjusted to 7.5. The cellulase was added to each sample in the test, according to DOE matrix (Table 1), and incubated at 50 °C for 2 h. Thirdly, the pH was adjusted to 7.5, and the protease was added to the test and incubated using the temperature and time described in Table 1. During protease hydrolysis, the pH was verified and adjusted to 7.5, when necessary.

The pretreatment and the two enzymatic hydrolyses were performed in an orbital shaker (Thermo Scientific™ MaxQ™ 6000) with an agitation of 125 rpm. The hydrolysis was finished by inactivating the enzymes at 90 °C for 10 min. Samples were centrifuged at 5000× *g* for 20 min, and the supernatant (henceforth called hydrolysate) was collected.

For the design of experiments (DOE), the hydrolysates were not freeze-dried. Thus, for protein quantification, the dry weight was determined, in duplicate, at 105 °C. Although not being used as responses for the DOE, the hydrolysate production yield (%) and protein release (%) were calculated for all 54 DOE experiments (Supplementary Materials). For the scaled-up process, the supernatant was freeze-dried for further analysis.

### 4.3. Experimental Design

A Box–Behnken experimental design was performed to determine the most influential factors potentiating the production of hydrolysates rich in proteins and bioactive properties from the microalgae *C. vulgaris*. The four factors evaluated were protease hydrolysis temperature (°C) (X_A_), cellulase % (X_B_), protease % (X_C_), and protease hydrolysis time (h) (X_D_). In previous studies, several cellulase percentages showed a potential effect on protein release. However, the ideal percentage was evaluated in the experimental design since antioxidant and antihypertensive properties were not assessed on the initial tests. Table 9 shows the established levels of the factors coded as −1 (low), 0 (central), and +1 (high). The selected responses were protein content (%), as well as antioxidant and antihypertensive potential. The DOE resulted in an arrangement of 27 treatments performed in duplicate (a total of 54 runs). Each hydrolysis was performed as described in Section 4.2.

#### Statistical Analysis and Statistical Model

The DOE matrix generation and the optimization analyses were performed using the software Statgraphic Centurion 19^®^. To compare the hydrolysis efficiency, a significance level of 5% (*p* < 0.05) was used. For the optimization, the nonsignificant effects were removed, and the responses (Y) were adjusted to the following second-order polynomial model (Equation (4)):

Y = β_0_ + β_A_X_A_ + β_B_X_B_ + β_C_X_C_ + β_D_X_D_ + β_A,B_X_A_X_B_ + β_A,C_X_A_X_C_ + β_A,D_X_A_X_D_ + β_B,C_X_B_X_C_ + β_B,D_X_B_X_D_ + β_C,D_X_C_X_D_ + β_A,A_X_A_^2^ + β_B,B_X_B_^2^ + β_C,C_X_C_^2^ + β_D,D_X_D_^2^ + ε, (4)

where Y is the measured response, β_0_ is a constant, β_A_, β_B_, β_C_, and β_D_ are the regression coefficients associated with linear effects of the variables X_A_, X_B_, X_C_, and X_D_, β_A_^2^–β_D_^2^ correspond to the quadratic effect, β_A,B_–β_C,D_ represent the coefficients for the interaction effects, and ε is the residual error. For the final models of each response, only the significant effects were considered (*p* < 0.05). A Derringer’s desirability function was applied to the results of each design, to optimize the multiple responses obtained (Suich and Derringer, 1980).

### 4.4. Protein Quantification

The micro-Kjeldahl method was used for determining the total nitrogen content of each hydrolysate as described before [25]. For the 54 hydrolysates resulting from the DOE, 1.0 mL of the sample was used, and the dry weight was used to achieve the protein content (%) per g of dry hydrolysate. For the scaled-up samples, 0.2 g of the freeze-dried hydrolysate was digested. All the analyses were performed in triplicate. Equations (5) and (6) were used to determine each sample’s total nitrogen and protein percentage.

(5) $${\mathrm{Total}\;\mathrm{nitrogen}\;(\%)=f\;\times \;(V}_{\mathrm{sample}}{\;-\;V}_{\mathrm{blank}})\;\times \;\left(\frac{100}{\mathrm{Sample}\;\mathrm{weight}}\right),$$

(6) Protein content (%)=Total nitrogen (%) × 6.25,

where f (HCl 0.1 M) = 0.0014, and 6.25 corresponds to the nitrogen to protein conversion factor, as described before for *C. vulgaris* protein determination [40,41].

### 4.5. Analysis of Antioxidant Activity

The oxygen radical absorbance capacity (ORAC) assay was used to evaluate the hydrolysate’s antioxidant potential. ORAC was performed in a black 96-well microplate (Nunc, Denmark) according to the method described by Coscueta et al. (2020) [42]. All the hydrolysates were analyzed in triplicate. The reaction was performed in a multi-detection plate reader (Synergy H1; BioTek Instruments, Winooski VT, USA) with excitation and emission wavelengths of 485 nm and 528 nm, respectively. Trolox (1–8 µM, final concentration in well) was used as the standard for the calibration curve. The results were expressed in µmol TE (Trolox equivalent)/mL for DOE hydrolysate or in µmol TE/g hydrolysate and µmol TE/g protein for scaled-up hydrolysate.

### 4.6. Analysis of the Antihypertensive Potential

The antihypertensive potential of the hydrolysates was evaluated by their ability to inhibit the angiotensin I-converting enzyme (iACE). The iACE assay was performed in a black 96-well microplate (Nunc, Denmark) according to a method described previously [43]. The fluorometric reactions occurred in a multi-detection plate reader (Synergy H1; BioTek Instruments, Winooski, VT, USA), controlled by the Gen5 BioTek software (version 3.04). All the samples were analyzed in triplicate.

For the DOE, iACE results were expressed as percentage inhibition. For the scaled-up hydrolysates, the concentration able to inhibit 50% of the ACE activity (IC_50_) was determined.

### 4.7. Analysis of the Antidiabetic Potential

The antidiabetic potential of the hydrolysate was evaluated by its ability to inhibit the enzyme α-glucosidase (EC 3.2.1.20). The reaction was carried out in a 96-well microplate, according to a method described previously [44]. The sample was evaluated at a concentration of 30 mg/mL. Acarbose (10 mg/mL) was used as a positive control. The sample and the positive control were dissolved in phosphate buffer (pH 6.9). For the negative control, phosphate buffer was used instead of the sample.

Briefly, 50 µL of sample or acarbose was mixed with 100 µL of phosphate buffer (0.1 M; pH 6.9) containing α-glucosidase solution (1.0 U/mL). The microplate was incubated at 25 °C for 10 min. Then, 50 µL of the substrate, a solution of *p*-nitrophenyl-α-d-glucopyranoside (5 mM in 0.1 M PBS (pH 6.9)), was added to each well. The reaction was performed for 5 min at 37 °C on a microplate reader (Synergy H1; BioTek Instruments, Winooski, VT, USA). The absorbance was registered at 405 nm at the beginning and end of the reaction.

The inhibitory ability was calculated following Equation (7) and expressed as percentage inhibition.

(7) $$\alpha -\mathrm{Glucosidase}\;\mathrm{inhibition}\;\left(\%\right)=\left[\left(\frac{{\Delta \mathrm{Abs}}_{\mathrm{control}}-{\;\Delta \mathrm{Abs}}_{\mathrm{sample}}}{{\Delta \mathrm{Abs}}_{\mathrm{control}}}\right)\right]\times \;100,$$

where ΔAbs_control_ and Δabs_sample_ correspond to the absorbance variation of the negative control and the sample, respectively.

### 4.8. Analysis by Size Exclusion Chromatography

The optimized *C. vulgaris* hydrolysate molecular weight (MW) distribution was determined using the AKTA Pure 25 system (GE Healthcare Life Sciences, Freiburg, Germany) coupled with two gel filtration columns (Superdex 200 increase10/300 G L and Superdex peptide, 10/300 G L) and controlled by UNICORN 7.0 software (GE Healthcare Life Sciences, Freiburg, Germany), as described previously [45]. A solution of 25 mM phosphate buffer (pH 7.0), 150 mM sodium chloride, and 0.2 g/L sodium azide was used as the mobile phase. An eluent flow of 0.5 mL/min and an absorbance of 280 nm were used to build samples and standard chromatograms.

To set up a standard curve, the following proteins and peptides, with known MW, were used: thyroglobulin (669 kDa), ferritin (440 kDa), aldolase (158 kDa), conalbumin (75 kDa), ovalbumin (43 kDa), carbonic anhydrase (29 kDa), ribonuclease A (13.7 kDa), aprotinin (6.5 kDa), and the antihypertensive peptide KGYGGVSLPEW (1.2 kDa). The hidden peak analysis was performed using software OriginPro 2021 v9.8.0.200.

## 5. Conclusions

In this work, we reported a sustainable methodology to obtain a natural hydrolysate from the microalga *C. vulgaris*, using sequential hydrolysis steps. A design of experiments allowed us to understand the more influencing factors on protein content, as well as antioxidant and antihypertensive potential. Additionally, the DOE permitted establishing the optimal hydrolysis conditions.

The hydrolysis performed using the optimal conditions produced a bioactive hydrolysate (yield: 61%) with 45% protein/peptides and great antioxidant (ORAC: 1035 µmol TE/g protein) and iACE (IC_50_: 286 µg protein/mL) values. These characteristics show the high commercial potential of the hydrolysate, since it can be used as an ingredient for the food, nutraceutical, and cosmetic industries. Future studies are necessary to confirm peptide bioavailability in the gastrointestinal tract and skin permeation.

## Figures and Tables

**Table 1:** Composition of the used *C. vulgaris* nutrients and pigments.

**Nutrients**	**Content (g/100 g)**
Protein	52.2
Fat	7.9
Carbohydrates	10.9
Dietary fiber	15.5
Ash	11.1
Moisture	2.4
**Pigments**	**Content (mg/100 g)**
Chlorophyll	1533
Total carotenoids	258

Data provided by the producer AllMicroalgae.

**Table 2:** Box–Behnken factorial design matrix for four factors and three responses.

Run		Factors			Response ^1^		
	Hydrolysis Temperature (°C) (X_A_)	% Cellulase (X_B_)	% Protease (X_C_)	Hydrolysis Time (h) (X_D_)	Protein Content (%)	ORAC (µmol TE/mL)	iACE (%) ^2^
1	50	1.67	3.33	2	56.58 ± 0.001	31.95 ± 0.03	58.20 ± 0.20
2	40	3.33	1.67	4	48.42 ± 0.02	28.09 ± 0.05	45.85 ± 0.05
3	50	3.33	3.33	4	52.48 ± 0.16	24.43 ± 0.01	46.28 ± 0.18
4	50	5.00	3.33	2	60.31 ± 0.05	30.12 ± 0.003	58.08 ± 0.10
5	40	5.00	3.33	4	55.19 ± 0.32	29.44 ± 0.03	56.99 ± 0.01
6	50	3.33	1.67	6	84.46 ± 0.17	9.24 ± 0.004	51.29 ± 0.01
7	50	5.00	3.33	6	53.92 ± 0.11	28.38 ± 0.002	60.71 ± 0.01
8	60	3.33	1.67	4	70.44 ± 0.25	26.45 ± 0.01	44.46 ± 10.71
9	40	3.33	5.00	4	54.67 ± 0.08	19.04 ± 0.003	55.94 ± 0.01
10	60	1.67	3.33	4	61.64 ± 0.09	24.84 ± 0.04	62.21 ± 0.01
11	50	3.33	3.33	4	55.41 ± 0.02	31.41 ± 0.02	63.59 ± 0.01
12	60	5.00	3.33	4	58.56 ± 0.29	19.40 ± 0.01	52.74 ± 0.06
13	50	3.33	1.67	2	57.24 ± 0.10	20.81 ± 0.02	42.85 ± 0.01
14	50	5.00	1.67	4	51.68 ± 0.04	14.38 ± 0.02	50.94 ± 0.01
15	40	3.33	3.33	6	67.57 ± 0.19	17.71 ± 0.07	48.17 ± 2.44
16	50	3.33	3.33	4	50.23 ± 0.03	28.40 ± 0.003	41.22 ± 0.01
17	40	3.33	3.33	2	63.89 ± 0.01	29.34 ± 0.02	35.17 ± 0.01
18	50	3.33	5.00	6	56.00 ± 0.21	24.81 ± 0.02	32.80 ± 0.01
19	40	1.67	3.33	4	65.61 ± 4.22	28.25 ± 0.05	40.84 ± 0.01
20	50	3.33	5.00	2	54.94 ± 0.02	28.45 ± 0.01	32.64 ± 0.01
21	60	3.33	5.00	4	64.37 ± 0.05	24.90 ± 0.10	56.61 ± 0.01
22	50	1.67	3.33	6	61.19 ± 0.18	27.53 ± 0.02	46.20 ± 0.20
23	60	3.33	3.33	2	67.06 ± 0.54	17.10 ± 0.10	39.95 ± 0.05
24	50	1.67	5.00	4	51.56 ± 0.19	30.41 ± 0.05	48.26 ± 0.04
25	60	3.33	3.33	6	60.53 ± 2.63	21.34 ± 0.08	53.00 ± 0.01
26	50	5.00	5.00	4	47.62 ± 0.17	27.66 ± 0.06	44.03 ± 0.01
27	50	1.67	1.67	4	53.38 ± 0.13	15.73 ± 0.02	38.00 ± 0.004

^1^ Values expressed as mean ± SD of two replicates; ^2^ ACE inhibitory activity measured in 0.5 mg hydrolysate/mL.

**Table 3:** Analysis of variance (ANOVA) for protein percentage.

Model	Sum of Squares	DF	Mean Square	F-Value
X_B_ (Cellulase)	45.7915	1	45.7915	144.25
X_C_ (Protease)	26.9145	1	26.9145	84.78
X_A_^2^	458.333	1	458.333	1443.81
X_A_X_B_	7.25992	1	7.25992	22.87
X_A_X_C_	484.661	1	484.661	1526.75
X_A_X_D_	23.8453	1	23.8453	75.12
X_B_X_C_	2.5088	1	2.5088	7.90
X_B_X_D_	60.4451	1	60.4451	190.41
X_C_^2^	7.49445	1	7.49445	23.61
X_D_^2^	352.242	1	352.242	1109.61
*R*^2^ = 98.50, Adj-*R*^2^ = 98.04, CV = 0.56				

**Table 4:** Analysis of variance (ANOVA) for ORAC.

Model	Sum of Squares	DF	Mean Square	F-Value
X_A_ (Temperature)	285.086	1	285.086	156.48
X_B_ (Cellulase)	9.98998	1	9.98998	5.48
X_C_ (Protease)	576.474	1	576.474	316.41
X_D_ (Time)	137.904	1	137.904	75.69
X_A_^2^	209.962	1	209.962	115.24
X_A_X_B_	21.9784	1	21.9784	12.06
X_A_X_C_	380.681	1	380.681	208.95
X_A_X_D_	125.928	1	125.928	69.12
X_B_^2^	31.8683	1	31.8683	17.49
X_C_^2^	393.421	1	393.421	215.94
X_C_X_D_	31.5217	1	31.5217	17.30
X_D_^2^	11.9879	1	11.9879	6.58
*R*^2^ = 88.66, Adj-*R*^2^ = 85.08, CV = 1.35				

**Table 5:** Analysis of variance (ANOVA) for iACE.

Model	Sum of Squares	DF	Mean Square	F-Value
X_A_ (Temperature)	112.839	1	112.839	4.57
X_B_ (Cellulase)	147.757	1	147.757	5.98
X_D_ (Time)	106.64	1	106.64	4.31
X_A_ X_B_	328.448	1	328.448	13.29
X_B_^2^	103.358	1	103.358	4.18
X_B_ X_C_	147.662	1	147.662	5.97
X_B_ X_D_	107.018	1	107.018	4.33
X_C_^2^	443.049	1	443.049	17.93
X_D_^2^	210.548	1	210.548	8.52
*R*^2^ = 42.86, Adj-*R*^2^ = 30.90, CV = 4.97				

**Table 6:** Single and multiple response optimization *.

Factors	Response			Multiple Responses
	Protein	ORAC	iACE	
Temperature (°C)	59.9	40.0	40.0	40.0
Cellulase (%)	5.0	5.0	5.0	5.0
Protease (%)	1.7	2.9	2.7	3.9
Time (h)	2.0	2.0	5.3	2.3

* Optimal conditions predicted by the experimental design to maximize protein release and antioxidant and antihypertensive activities of the hydrolysate, as well as for multiple optimization.

**Table 7:** Results obtained in scaled-up extractions, performed in triplicate, with the optimal conditions described in Table 6.

Evaluated Characteristics	Obtained Results
Bioactive hydrolysate yield (%)	61 ± 0.5
Protein (%)	45 ± 1.7
ORAC (µmol TE/g hydrolysate)	463 ± 39.9
ORAC (µmol TE/g protein)	1035 ± 68.7
iACE (IC_50_ µg of protein/mL)	286 ± 55.0
α-Glucosidase inhibition (%) (30 mg hydrolysate/mL)	31 ± 3.9

**Table 8:** Examples of alge and cyanobacteria hydrolysates/extracts regarding bioactive properties.

Specie	Hydrolysate/Extract Production	Antioxidant Activity	iACE (IC50)	α-Glucosidase Inhibition	Ref.
*Bifurcaria bifurcata*	Ultrasound-assisted extraction using water/ethanol	556.20 µmol TE/g DW			[28]
*Chlorella vulgaris*	Ultrasound-assisted extraction using water/ethanol	31.21 µmol TE/g DW			[28]
	Enzymatic hydrolysis (pepsin)		29.6 µM		[35]
*Chlorella ellipsoidea*	Enzymatic hydrolysis (alcalase)		128.4 µM		[36]
*Fucus spiralis*	Enzymatic hydrolysis (cellulase and bromelain)		0.5–2.0 mg/mL		[37]
*Chlorella pyrenoidosa*				68.28% (1 mg/mL)	[34]
*Porphyra dioica*	Alcalase and Flavourzyme	3.14 µM TE/µM peptide	163.6 µM		[38]

**Table 9:** Levels of foir experimental factors for the experimental design.

Factors	Levels		
	Low (−1)	Central (0)	High (+1)
Temperature (X_A_)	1.67	3.33	5.00
% Cellulase (X_B_)	1.67	3.33	5.00
% Protease (X_C_)	40	50	60
Time (h) (X_D_)	2	4	6

## Data Availability

Not applicable.

## Supplementary Materials

The following supporting information can be downloaded at https://www.mdpi.com/article/10.3390/molecules27082505/s1: Figure S1. Protein concentration of the extract after treatment with an acid solution, or after hydrolysis with cellulase, or after being submitted to a combination of both conditions, after 30 and 60 min. Different letters refer to significant differences at *p* < 0.05 (ANOVA One-Way followed by Tukey’s post hoc test); Figure S2. Protein concentration of the extract after being treated with different % of an acid solution for 1 h, followed by a cellulase hydrolysis. Different letters refer to significant differences at *p* < 0.05 (ANOVA One-Way followed by Tukey’s post hoc test);Figure S3. Protein concentration of the extract after being incubated with a 2% acid solution, followed by a cellulase hydrolysis at different temperatures. Different letters refer to significant differences at *p* < 0.05 (ANOVA One-Way followed by Tukey’s post hoc test); Figure S4. Protein concentration of the extract produced by treatment with a 2% acid solution, followed by a hydrolysis with 2% cellulase. Different letters refer to significant differences at *p* < 0.05 (ANOVA One-Way followed by Tukey’s post hoc test); Table S1. Box-Behnken factorial design matrix for four factors and hydrolysate yield and protein release for each run; Table S2. Hidden peaks retention volume, resulting from the model Local Maximum.
