# Supplementary material for: Bioactive Hydrolysates from Chlorella vulgaris: Optimal Process and Bioactive Properties
Source: Molecules. 2022 Apr 13;27(8):2505. doi: 10.3390/molecules27082505 (PMC9026812; doi:10.3390/molecules27082505)
Supplement: Supplementary file 1 [file molecules-27-02505-s001.zip › molecules-1665560-supplementary.pdf]

Supplementary Materials

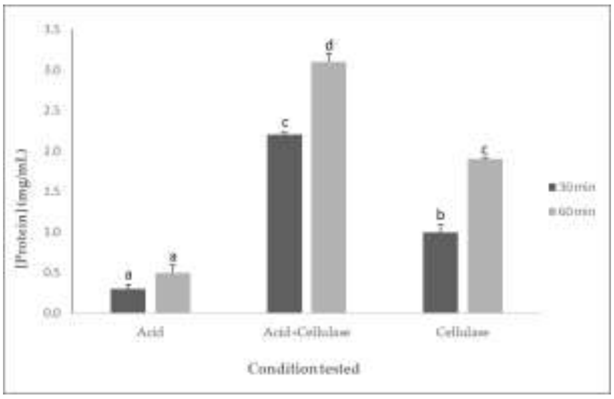

Figure S1. Protein concentration of the extract after treatment with an acid solution, or after hydrolysis with cellulase, or after being submitted to a combination of both conditions, after 30 and 60 min. Different letters refer to significant differences at $p<0.05$ (ANOVA One-Way followed by Tukey's post hoc test).

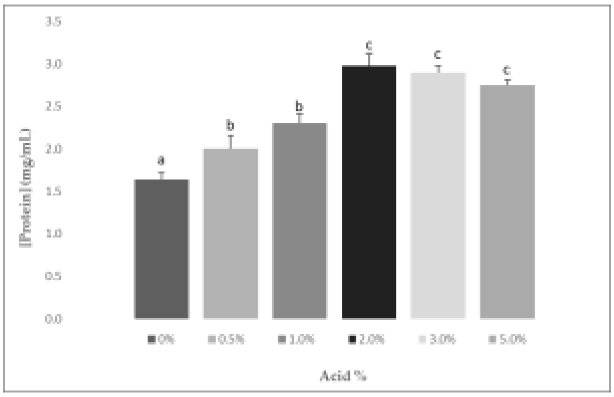

Figure S2. Protein concentration of the extract after being treated with different % of an acid solution for 1 h, followed by a cellulase hydrolysis. Different letters refer to significant differences at $p<0.05$ (ANOVA One-Way followed by Tukey's post hoc test).

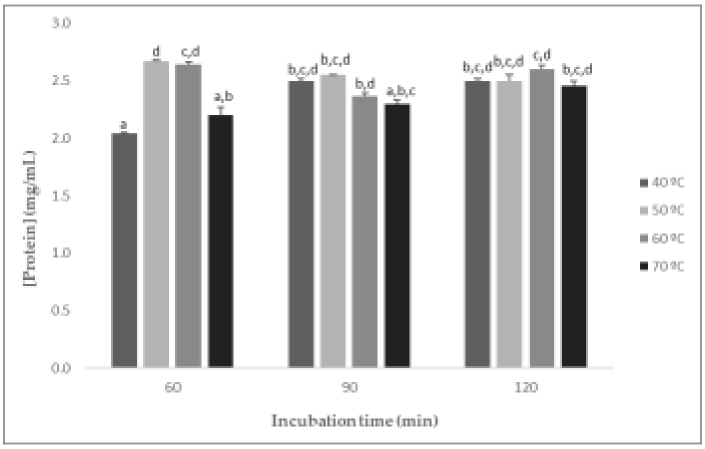

Figure S3. Protein concentration of the extract after being incubated with a 2% acid solution, followed by a cellulase hydrolysis at different temperatures. Different letters refer to significant differences at $p<0.05$ (ANOVA One-Way followed by Tukey's post hoc test).

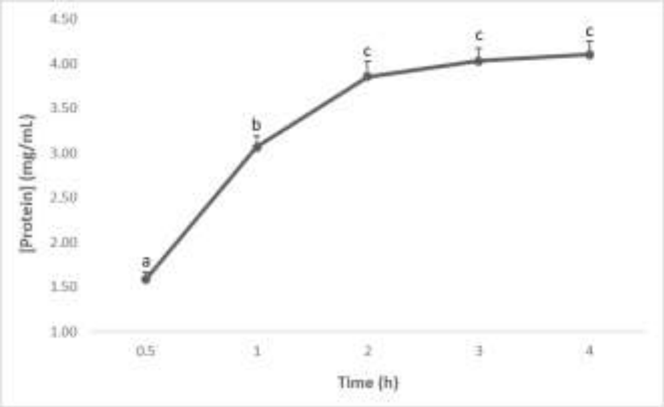

Figure S4. Protein concentration of the extract produced by treatment with a 2% acid solution, followed by a hydrolysis with 2% cellulase. Different letters refer to significant differences at $p<0.05$ (ANOVA One-Way followed by Tukey's post hoc test).

Table S1. Box-Behnken factorial design matrix for four factors and hydrolysate yield and protein release for each run.

Run	Factors				Evaluated characteristics	
	Hydrolysis temperature (ºC) (X_A)	% Cellulase (X_B)	% Protease (X_C)	Hydrolysis time (h) (X_D)	Hydrolysate yield (%)	Protein release (%)
1	50	1.67	3.33	2	69.21 ± 0.01	74.08 ± 0.01
2	40	3.33	1.67	4	60.98 ± 0.02	55.65 ± 0.32
3	50	3.33	3.33	4	72.04 ± 0.24	71.39 ± 0.54
4	50	5.00	3.33	2	68.12 ± 0.12	77.91 ± 0.20
5	40	5.00	3.33	4	66.63 ± 0.23	69.56 ± 0.17
6	50	3.33	1.67	6	47.52 ± 0.12	76.07 ± 0.03
7	50	5.00	3.33	6	76.02 ± 0.02	77.60 ± 0.18
8	60	3.33	1.67	4	60.04 ± 0.24	79.42 ± 0.45
9	40	3.33	5.00	4	58.95 ± 0.05	60.65 ± 0.63
10	60	1.67	3.33	4	60.74 ± 0.14	70.47 ± 0.27
11	50	3.33	3.33	4	72.30 ± 0.10	75.70 ± 0.41
12	60	5.00	3.33	4	52.20 ± 0.00	57.38 ± 0.12
13	50	3.33	1.67	2	47.31 ± 0.09	50.63 ± 0.10
14	50	5.00	1.67	4	47.00 ± 0.13	45.89 ± 0.25
15	40	3.33	3.33	6	50.35 ± 0.01	64.01 ± 0.35
16	50	3.33	3.33	4	72.39 ± 0.05	68.70 ± 0.14
17	40	3.33	3.33	2	49.25 ± 0.20	58.95 ± 1.21
18	50	3.33	5.00	6	58.40 ± 3.18	61.66 ± 2.52
19	40	1.67	3.33	4	49.18 ± 0.00	58.99 ± 0.85
20	50	3.33	5.00	2	68.80 ± 0.07	71.04 ± 1.32
21	60	3.33	5.00	4	67.76 ± 0.42	82.13 ± 0.12
22	50	1.67	3.33	6	61.67 ± 0.25	71.19 ± 0.84
23	60	3.33	3.33	2	55.38 ± 2.90	69.37 ± 1.21
24	50	1.67	5.00	4	73.35 ± 0.06	71.05 ± 1.48
25	60	3.33	3.33	6	67.10 ± 0.03	76.08 ± 1.45
26	50	5.00	5.00	4	74.54 ± 1.20	66.54 ± 0.83
27	50	1.67	1.67	4	55.57 ± 0.42	56.22 ± 0.15

Table S2. Hidden peaks retention volume, resulting from the model Local Maximum.

Peak number	Retention volume (ml)
1	25.70
2	25.80
3	25.83
4	25.86
5	25.89
6	25.92
7	25.95
8	25.97
9	26.02
10	26.24
11	26.33
12	26.45
13	26.48
14	26.58
15	26.64
16	26.66
17	26.70
18	26.75
19	26.78
20	26.80
21	26.81
22	26.83
23	26.86
24	26.99
25	27.05
26	27.26
27	27.37
28	27.41
29	27.58
30	27.87
31	27.89
32	28.01
33	28.19
34	28.49
35	36.39
36	36.41
37	38.16
38	41.32
39	43.22
40	49.43
41	49.44
42	53.19
